# Linked Registries: Connecting Rare Diseases Patient Registries through a Semantic Web Layer

**DOI:** 10.1155/2017/8327980

**Published:** 2017-10-29

**Authors:** Pedro Sernadela, Lorena González-Castro, Claudio Carta, Eelke van der Horst, Pedro Lopes, Rajaram Kaliyaperumal, Mark Thompson, Rachel Thompson, Núria Queralt-Rosinach, Estrella Lopez, Libby Wood, Agata Robertson, Claudia Lamanna, Mette Gilling, Michael Orth, Roxana Merino-Martinez, Manuel Posada, Domenica Taruscio, Hanns Lochmüller, Peter Robinson, Marco Roos, José Luís Oliveira

**Affiliations:** ^1^University of Aveiro, DETI/IEETA, Aveiro, Portugal; ^2^Galician Research and Development Center in Advanced Telecommunications (GRADIANT), Pontevedra, Spain; ^3^National Center for Rare Diseases, Istituto Superiore di Sanità, Rome, Italy; ^4^Leiden University Medical Centre (LUMC), Leiden, Netherlands; ^5^International Centre for Life, Institute of Genetic Medicine, Newcastle University, Newcastle upon Tyne, UK; ^6^Research Programme on Biomedical Informatics (GRIB), Hospital del Mar Medical Research Institute (IMIM), Universitat Pompeu Fabra (UPF), Barcelona, Spain; ^7^Institute of Rare Diseases Research, ISCIII, SpainRDR and CIBERER, Madrid, Spain; ^8^John Walton Muscular Dystrophy Research Centre, Institute of Genetic Medicine, Newcastle University, Newcastle upon Tyne, UK; ^9^The European Huntington's Disease Network, University Hospital of Ulm, Ulm, Germany; ^10^Department of Neurology, University Hospital of Ulm, Ulm, Germany; ^11^Karolinska Institutet, Solna, Sweden; ^12^Institute of Medical Genetics, Charité Universitätsmedizin Berlin, Berlin, Germany

## Abstract

Patient registries are an essential tool to increase current knowledge regarding rare diseases. Understanding these data is a vital step to improve patient treatments and to create the most adequate tools for personalized medicine. However, the growing number of disease-specific patient registries brings also new technical challenges. Usually, these systems are developed as closed data silos, with independent formats and models, lacking comprehensive mechanisms to enable data sharing. To tackle these challenges, we developed a Semantic Web based solution that allows connecting distributed and heterogeneous registries, enabling the federation of knowledge between multiple independent environments. This semantic layer creates a holistic view over a set of anonymised registries, supporting semantic data representation, integrated access, and querying. The implemented system gave us the opportunity to answer challenging questions across disperse rare disease patient registries. The interconnection between those registries using Semantic Web technologies benefits our final solution in a way that we can query single or multiple instances according to our needs. The outcome is a unique semantic layer, connecting miscellaneous registries and delivering a lightweight holistic perspective over the wealth of knowledge stemming from linked rare disease patient registries.

## 1. Introduction

A rare disease is a particular health condition affecting at most 1 in 2000 patients [1]. According to the Orphanet inventory (http://www.orpha.net/), there are approximately 6000 to 8000 rare diseases, from which about 80% have a genetic origin. Complex health implications behind rare diseases are seldom considered in medical or social care. Due to the rarity of each individual disease and their often-complex nature, this group is underrepresented in research and treatment developments. At the patient level, the diagnosis of a rare disease generally means higher difficulty to find support, both clinical and psychological [2]. The existence of a small number of cases for each disease creates additional barriers in the translational research pathway, as it is difficult to identify and coordinate a substantial cohort [3, 4]. Nevertheless, altogether rare disease patients comprise an estimated 6 to 8% of the EU population [5]. During the last decade, several small disease-specific databases were developed, related, for instance, to neurological disorders or muscular problems [6]. While they provide high quality information and resources, their disease coverage is small and typically with a regional or national scope. To achieve higher statistical evidences, we need more extensive cohorts of patients with similar features, from worldwide population. Hence, discovering rare disease causing genes can have impact on all medical treatment stages, from clinical diagnostics to insights gained into biological mechanisms and common diseases [7].

In addition to long-term patient care improvements, understanding gene-disease associations is a fundamental goal for computational biology research, especially in rare disease where genotype-phenotype connections are typically limited to one or a few more genes [8, 9]. Moreover, it is in these particular conditions that the strongest relations between genotypes and phenotypes are identified. Hence, to fully understand the underlying causes of diseases, we need to connect knowledge that is widespread throughout miscellaneous registries.

Rare disease patient registries are typically fragmented by data type and disease. Furthermore, these systems have poor interoperability due to the high complexity and heterogeneity of data types and the lack of using standards on data model and data descriptions. Coupled with severe requirements to protect data, the access to the patient's registries is restricted, converting these valuable and distributed sources in closed data silos. This poses a barrier for linking patient-centric electronic records across registries and across diseases. To tackle these integration barriers, we started the study looking for relevant questions difficult to answer without an infrastructure of integrated patient data from several registries. The motivating questions were, for instance,

1. Given a set of phenotypes that are relevant for neuromuscular (e.g., DM, FSHD, LGMD2I) and neurodegenerative diseases (e.g., HD, Ataxia), can we find patients in a disease nonspecific way?
2. More specifically, based on “Ambulation”, “Age”, and “Country”, can we get the number of patients?

Answering this kind of questions requires data of disparate nature and from multiple sources. To harmonize these data into a semantic layer we need an integration platform capable of converting any data format into RDF. This implies abstracting registry concepts and their attributes, such as Patient (sex, birthday, and country); Disease; Phenotype (motor, ambulation); and Genetic Variation and then representing them into a graph data model in which the semantics of the objects and their relationships can be described with standard or widely adopted ontologies. Finally, patient registries data should be aggregated by concept. In each concept, data elements, or instances that represent the same entity but have different text mentions in each registry, must be mapped to an ontology term. For example, Orphanet Rare Disease Ontology (ORDO) for diseases and the Human Phenotype Ontology (HPO) for phenotypes can be used to make data interoperable and linkable in the Web of data. The use of domain-specific and commonly used ontologies adds value to data, through an integrated knowledge base that is searchable and comparable by user and by machines [10]. Furthermore, interlinking patient registry data with external linked datasets allows enriching the current knowledge in the rare disease research.

In this work, we have developed a new semantic layer on top of existing patient registries, to allow extracting anonymised data from the original datasets, translate them to a common shared exchange model, and make them available to the research community (available at http://bioinformatics.ua.pt/linked-registries-app/). The solution addresses three key requirements from the patient registries research community: (1) data model agnostic; (2) distributed and encapsulated; and (3) knowledge-oriented.

Firstly, data harmonization strategies are data model agnostic and work regardless of registries' data format and internal structure. This is clearly important as we are dealing with systems featuring assorted characteristics, from relational databases and service endpoints, up to Excel spreadsheets.

Next, the solution is distributed and encapsulated. When dealing with rare disease patients, it is imperative to ensure data anonymity and privacy. Hence, we need tools that extract meaningful data while maintaining hidden all the attributes that may disclose patients' identification.

At last, our approach takes advantage of Semantic Web technologies to improve how we publish, access, express, and share knowledge across the Web. From a technological perspective, the system was built on top of COEUS [11], an application framework that streamlines data integration with semantic representation. As patient registries are shared within this platform, researchers and developers are able to perform federated queries, covering miscellaneous databases, just like they would query a single local dataset.

In summary, we explore a Semantic Web approach and a nonintrusive strategy to interconnect, enrich, and federate data from multiple patient registries, allowing extending the knowledge behind these distributed repositories.

## 2. Background

### 2.1. Patient Registries

The collection and maintenance of patient registers have been assuming a key role in the identification of new treatments and in the improvement of care. In particular, personal genetic records are of growing interest. These data are increasingly important for diagnosis, resolution, and therapeutic treatment of rare diseases. Hence, databases with information about human genome, such as the Human Gene Mutation Database (HGMD) [12] or the 1000 Genomes Project [13], currently have a growing relevance. Moreover, it is important to reuse these data in novel biomedical software to enable its usage on daily medical workflows.

The value of individual data increases when it is aggregated and presented in a unified way, both for humans and computers [14]. Orphanet provides a public portal, for professionals and patients, with the most updated information about rare diseases and orphan drugs [15]. It also displays information on specialized consultations, diagnostics, research projects, clinical trials, and support groups. Diseasecard [16] is another platform that aggregates genotype-to-phenotype information regarding rare diseases, pointing to key elements for both the education and the biomedical research field. While these systems do not provide repositories for patient-level data, they are useful resources for sharing and disseminating existing knowledge and expertise.

Besides the important role of these specialized repositories, the integration of knowledge that can be extracted from distinct electronic health records (EHRs) is also a major challenge to support personalized medicine. Data from gene sequences, mutations, proteomics, and drug interactions (the genotype) can now be combined with data from EHRs, medical imaging, and disease-specific information stored in patient registries (the clinical phenotype). Hence, it is crucial to start exploring patient-level data from rare diseases registries, which often include personal data, diagnosis, clinical features, phenotypes, genotypes, treatments, and clinical follow-up.

According to Orphanet, there are over 600 rare disease registries just in Europe, with different aims and objectives, with access to different resources and collecting different datasets. Registries have traditionally been developed to accelerate the translational research pathway helping to move therapies from bench to bedside as quickly as possible. They provide a tool for the feasibility and planning of clinical trials as well as means to identify and recruit patients into research. However, the purpose and utility of registries has a much broader reach providing a source of natural history data and a basis for hypothesis generation that can advance research into a given field.

A single academic can set up a registry or a clinician with an interest in a particular field, or a disease network, or—as is increasingly common—a patient organization. The variety in origin explains the variety of funding schemes (sustainability models) and data collection techniques [17].

The highly developed registries (e.g., Cystic Fibrosis [18, 19]) act as detailed natural history studies, with data collected at fixed time points in the clinical setting and stored in bespoke software solutions. However, many registries are online self-report systems, with patients entering data through a Web portal. There are also examples of a combined approach: patients initiate registration, while physicians verify details through the same Web portal. This disparity in data collection increases the complexity of a unified system. The data items themselves are not standardized across all rare diseases though a significant amount of effort has been applied in this area. Some consensus has been reached in certain disease areas, such as Duchenne Muscular Dystrophy (DMD), where a federated registry system exists under the umbrella of TREAT-NMD (http://www.treat-nmd.eu/) [20, 21].

One step towards harmonization can come in the form of international medical classifications or languages, such as UMLS [22] or SNOMED-CT [23], among some other terminologies, and, more recently, the use of phenotype ontologies, such as HPO [24], has been proposed as a tool for phenotype standardization. Ontologies are structured representation of knowledge using a standardized, controlled vocabulary for data integration, organization, searching, and analysis. The use of ontologies to identify and annotate data in patient registries ensures interoperability and access, and it also enables cross-cohort comparisons and filtering. Importantly, it allows the development of new bioinformatics tools, covering the automated and systematic matching of clinically similar representations of phenotypes to assist in differential diagnosis, among others.

These patient-centric databases offer unique specialized views over their internal datasets. However, while there are huge amounts of data scattered throughout multiple stakeholders, they are wildly difficult to obtain or access. The main reasons are the lack of semantic compatibility and the evident low motivation of data owners to share and spread their valuable datasets and, thus, individual efforts remain isolated. This is a critical hindering factor in rare disease research, where a sole centre may collect only a small number of patients with a certain disease. The outcome of this is that, in the end, there is not enough data to generate statistically meaningful conclusions. As such, we cannot discover or infer new knowledge because there is no access to a minimal amount of patient data.

To cope with these challenges we need a platform that offers a unique holistic view promoting the collaboration of multiple entities towards the study of rare diseases and assessment of patients' evolution [25]. According to our study, only one related exchange platform was developed regarding the rare diseases domain: The Matchmaker Exchange [26]. This platform provides a systematic approach to create a federation network of genotypes and phenotypes databases through a common application programming interface (API). This helps in the process of finding common genotype/phenotype pairs in multiple individuals. However, this approach requires deposition of the data into the main database or the setup of local instances, always ensuring a set of services and end users agreements. Comparatively, our proposed solution is more generic and suitable for the creation of independent systems that can be plugged into any existing patient registry without changing it. Furthermore, as our system relies on Semantic Web technologies and standards, this will promote a better translation, federation, and discovery of new knowledge. By exploring the Matchmaker system (and its different aims), we believe that our approach is a better milestone towards semantic interoperable rare diseases knowledge.

### 2.2. Semantic Web

The Semantic Web arises as a ground breaking paradigm to foster the intelligent integration of structured information. Sustained by state-of-the-art standards such as RDF, OWL, SPARQL, and LinkedData, the Semantic Web promotes better strategies to express, infer, and make knowledge interoperable.

Latest advances in the area cover the research and development of new algorithms to further improve how we collect data, transform data into meaningful knowledge assertions, and publish connected knowledge. State-of-the-art solutions, including the EBI RDF Platform [27], COEUS [11], or SADI [28], pave the way towards interoperable scientific knowledge. From a large-scale perspective, we can now see the Semantic Web as a single knowledge network. Available technologies foster data integration and publishing, enabling an effortless connection between heterogeneous distributed knowledge.

The true value behind Semantic Web technologies lies in on how easy it is to access and exchange knowledge between independent systems. The Linked Data guidelines, from the W3C working group, promote accessing data via unique URIs that, besides identifying knowledge, must resolve to real data. SPARQL, the Semantic Web query language, complements Linked Data.

Knowledge bases with an open SPARQL endpoint enable direct queries to their content. This empowers researchers and developers alike with an open knowledge highway. In this area, COEUS can play a fundamental role by delivering a “Semantic Web in a box” approach, enabling the rapid development of new knowledge management systems with Semantic Web technologies [29, 30]. COEUS allows gathering data from heterogeneous repositories and publishes them via SPARQL endpoint and Linked Data interfaces.

## 3. Methods

### 3.1. Architecture

Semantic data integration is, in itself, a complex data engineering issue if we have to code every component of the software solution [31, 32]. Leveraging on previous results [33], we use COEUS as the baseline framework of our platform. Exploring its flexible integration engine enables simplifying the overall platform architecture through the creation of a comprehensive dependency-based resource integration network.

**Figure 1:**
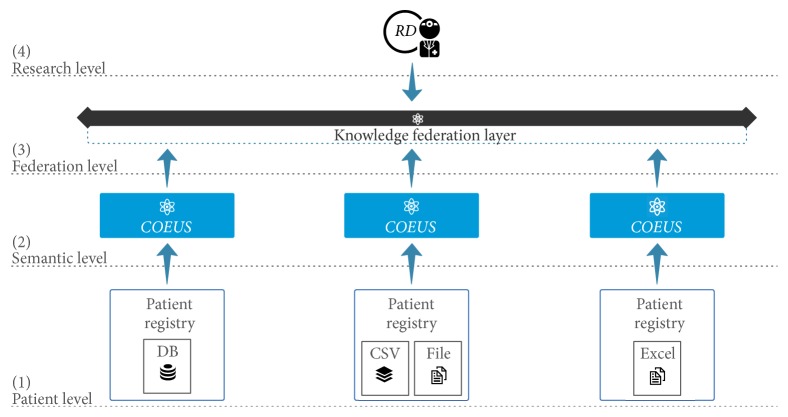
Knowledge federation architecture, integrating distributed patient registries via COEUS.

Figure 1 presents the platform's distributed architecture, which is organized in four levels: (1) Patient, (2) Semantic, (3) Federation, and (4) Research.

At the patient level we gather information from the distributed and heterogeneous patient registries, which can be stored in multiple formats using various technologies (e.g., relational databases, text files, and spreadsheets). Patient registries can be integrated in the framework regardless of their location and their quantity.

At the second level we include additional semantics to patient registries data. This is done using COEUS, which acts as the main abstraction, storage, and publishing engine. Here, we manage the anonymised patient data, translating from their primitive format to common biomedical ontologies.

The third level provides the knowledge federation and data exploration capabilities; that is, SPARQL queries can be forwarded to several patient registries endpoints. COEUS acts here as a middleware component between the patient registry triple store and the public knowledge federation layer.

Finally, at the upper level researchers can perform general queries that combine data from one or more patient registries. In a sense, query federation enables performing SQL-like UNIONs or JOINs across multiple knowledge bases. This empowers knowledge inference and reasoning queries to go beyond what is currently possible.

### 3.2. Workflow

**Figure 2:**
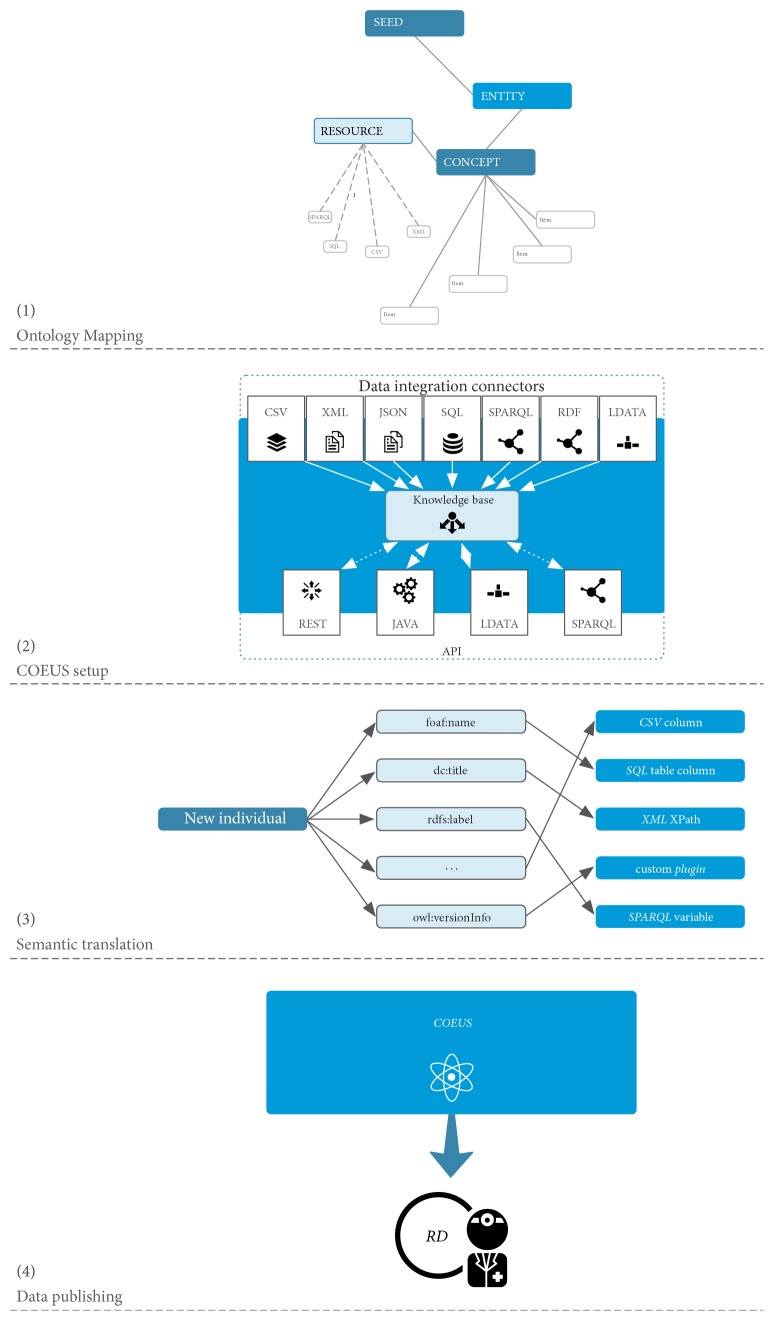
Simplified registry publication workflow.

Publishing anonymised patient registries data in a semantic way requires a comprehensive workflow.  Figure 2 describes the key steps in this semantic integration and translation pipeline: (1) ontology mapping; (2) COEUS setup; (3) semantic translation; and (4) data publishing.

The first step consists in defining the best ontologies to map common patient's data. HPO [24], UMLS [34], ICD [35], and ORDO [15] are the most widely used ontologies in the rare diseases field. One of the great advantages of using Semantic Web technologies is that any external ontology can be used to complement or extend COEUS internal model. As long as clinicians understand the new predicates, any number of properties can be included, semantically mapping concepts or entities to existing ontologies or adding further properties to describe entities or concepts. Moreover, we may combine multiple ontologies, that is, the same data element can be mapped to terms from more than one ontology, optimising its expressiveness and enriching the way it can be used in future research environments. In this step, semiautomated annotation tools such as SORTA [36] and EGAS [37] along human curation experts play an important role for the annotation of biomedical data (e.g., phenotypes, diseases).

The second step of the pipeline consists in the configuration and deployment of a new COEUS instance. The setup involves defining how data will be extracted and mapped into the selected ontology terms. Using COEUS connectors we have to specify where the data comes from (Excel, CSV, or XML files; SQL databases; or SPARQL/LinkedData endpoints) and how we will map it to the ontologies. For instance, for a patient registry available as a CSV file, we need to specify the file location and, for each mapped ontology term, the column containing the actual data elements.

In the following stage, the semantic translation process, knowledge base elements, and their data and object properties are created in real-time from the integrated data. This step elevates data in primitive formats to a new semantic abstraction level. The process is complete when all data are imported into a new triple store, making it available for external use through the various data publishing endpoints.

### 3.3. Implementation

**Figure 3:**
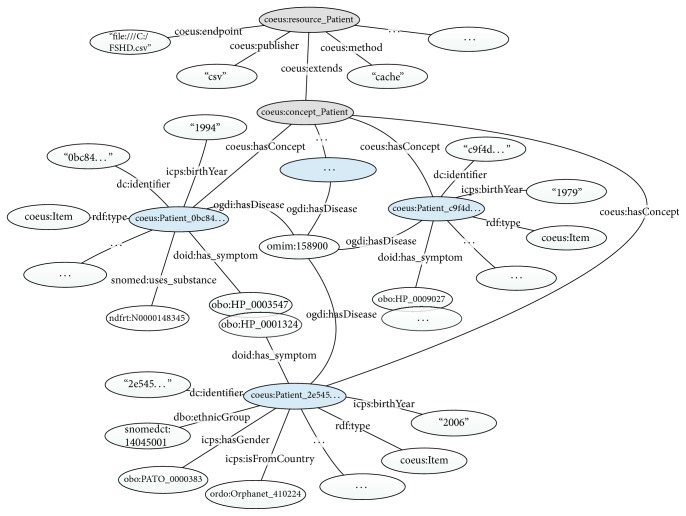
Patient registry model overview. Facioscapulohumeral Muscular Dystrophy patients share concepts and relationships, creating a fully connected network.

COEUS framework is focused on helping researchers in the construction and publishing process of new semantically enhanced systems. It offers a good starting point to integrate disparate data due to the advanced ETL (Extract-Transform-Load) processes in its engine. These algorithms facilitate the “triplification” process, in which all data are converted to a simple* subject-predicate-object* model. Moreover, it makes the integrated information available through a hierarchical model establishing relationships between data in an “Entity-Concept-Item” structure (e.g., Protein-Uniprot-P51587). To create each registry's knowledge base according to this organized model, we must fulfil some initial requirements. Essentially, there are three main steps to achieve the final solution: the first is data selection, the second is the data sources configuration, and last one is the data integration process. In the first step, we have applied a preselection process to all registries data. In this process, we select the desired information for our study avoiding sensitive data and considering only nonidentifiable data. Patient registries have a lot of information, but some are redundant and incomplete. Regarding that, applying this initial filtering process is vital to build a consistent database. This process was only possible due to the involvement of respective data owners that translated what their data means. In the final stage of this initial filtering process, we exported the files of the distinct registries. Each dataset was imported to a separated COEUS instance, as we plan to have four distributed systems. In the second step, we define the data sources attributes for each registry. This starts by creating one* Resource* (Figure 2, block 1—ontology mapping) in the knowledge base that contains the respective data elements such as* Endpoint* location (i.e., the registries* file location*),* Publisher* (i.e.,* CSV* file),* CSV Starting line* (i.e.,* 1* as the registries file has headers), and* Method* (i.e.,* cache* as we will load the entire file into the system). Additionally, for each* Resource*, a combination of parameters (i.e.,* Selectors*) must also be included to establish the mapping between the information to be extracted from the registry (e.g., for each column) and the respective formal ontology terms that connects it. For instance, we can make use of the Human Disease Ontology [38] term* has_symptom* to establish the connection between a Facioscapulohumeral Muscular Dystrophy (FSHD) patient and the identified symptoms:* coeus:Patient_X doid:has_symptom obo:HP_0001324* (i.e., muscle weakness.). Likewise, we link each patient to its respective identifier by creating a* Selector* that makes the linkage between the CSV first column (with the patient IDs) in the parameter* query* and the property* dc:identifier* from the Dublin Core Ontology [39]. Establishing all these mappings in the registries records makes the foundation of an interconnected network of relationships between patients and respective features possible. An overview of the knowledge base model is available in Figure 3, showing a simplified view of Facioscapulohumeral Muscular Dystrophy Type 1 (*omim:158900*) patients relationships.

After these configurations, the last process encompasses the automatic integration and semantic mapping of data sources. To expose data, we provide several interoperability features such as SPARQL endpoint and a Linked Data interface. The SPARQL endpoint works as a federated query system, in which we can perform complex queries across patient registries. The Linked Data interface provides easy access to the patient information through the Web Browser or similar applications.

All the described processes are managed through COEUS Web user interface that provides an easy-setup solution to the installation and configuration process.

## 4. Results

### 4.1. Exploring Rare Diseases Patient Registries

Neurodegenerative and neuromuscular diseases are among the most frequent of rare diseases, affecting the life and mobility of more than 500,000 patients and families in Europe (http://rd-neuromics.eu/). We used the proposed architecture for the integration of four patient registries in the neuromuscular and neurodegenerative disease area. These registries collected patient data from ten different countries (United Kingdom, Italy, Spain, Denmark, France, Netherlands, Sweden, Austria, United States, and Germany) and gather information related to four rare diseases: Myotonic Dystrophies (DM), Facioscapulohumeral Muscular Dystrophy (FSHD), Fukutin Related Protein (FKRP) related conditions (e.g., LGMD2I), and Huntington's Disease (HD).

To first guide the development of our solution and also, at the end, to allow its validation, several questions were initially elaborated:

1. Can we find more than ten male and ten female patients that share a set of phenotypes and live in different countries?
2. To build a trial/research data set, how many patients have the desired conditions/requirements for the study and live closest to the clinical/research setting?
3. What are the phenotypes associated with Myotonic Dystrophies (DM) and Facioscapulohumeral Muscular Dystrophy (FSHD) diseases?
4. Are there patients treated with different therapies diagnosed with the same disease?
5. Are there patients treated with the same therapy but diagnosed with different diseases?
6. Can we find patients diagnosed with a certain disease with different states of morbidity?
7. Are there patients with this specific set of phenotypes?
8. Are there patients sharing phenotypes and diagnosed with different neuromuscular and neurodegenerative diseases?

To find the answers for these questions researchers and clinicians need to look for patient data that are fragmented in different registries and need to combine data across registries and across diseases. Without an infrastructure of integrated patient registries, this is not a straightforward endeavour because each patient registry is designed, described, and technically implemented in a particular way, and data are not connected. This means that to answer whatever of these questions one will spend a considerable amount of time to understand each registry data model, to access and retrieve each registry's needed data, to aggregate all registries' data in a meaningful manner, and, finally, to query-answer over the harmonized data to extract the information. These are rather inefficient and infeasible propositions. Thus, to gain a complete view on a specific disease and patient population of interest, and to retrieve the desired information to answer these questions, the linking of registries data sets is an essential step. The described research questions involved the collaboration of data owners and database managers, which participated actively in the data selection and harmonization processes, and providing continuous feedback for the final solution.

### 4.2. The Linked Registries Solution

**Figure 4:**
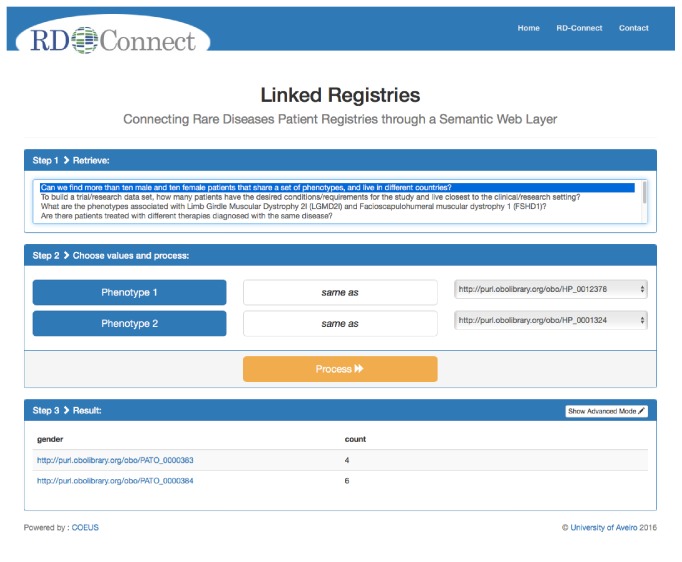
Linked Registries Web application interface.

The interconnection between disperse patient registries using COEUS facilitates our final solution in the way that we can query single or multiple instances according to our needs. However, to better access and enhance user interaction, we provide a single and Web based entry point to access the aggregated information available on each instance. This entry point is available at http://bioinformatics.ua.pt/linked-registries-app/ and is based on a combination of SPARQL federation queries templates with predefined variables. In each template question, variables can be adjusted according to the knowledge bases values automatically. All queries are generated in real-time by the application and can be edited or adjusted (by using the advanced mode) for a more accurate examination. By using this solution, users have the opportunity to find answers to the previously defined questions across disperse patient registries. For instance, to answer the first question we created a template federated query to retrieve results from all the registries. As we store patient's countries information in each COEUS instance using the same model, we are able to retrieve common characteristics from each instance. Regarding that, finding a cohort sharing a set of phenotype information and based in different countries can be done in a straightforward process (Figure 4).

In order to answer this type of question, we searched for male and female patients that have both “fatigue” (i.e., obo:HP_0012378) and “muscle weakness” (i.e., obo:HP_0001324) phenotypes. By querying our system, we retrieved male patients from six different countries, and female patients from four different countries. Therefore, for our particular set of registers, the number of patients (either in the male or female case) living in different countries that share those phenotypes is less than ten.

Concerning question (2), filtering patient characteristics according to some conditions, such as the use of gastric tube on a Myotonic dystrophy type 1 (MD1) patient, can be successfully performed. However, discovering patients living closest to their clinical/research setting is not a trivial task to perform due to the limitation of our registries' data, which only covers countries related information.

To answer inquiries similar to question (3) is also feasible. For instance, we can query the two remote databases (e.g., FKRP and FSHD) through their SPARQL endpoint and search for all shared phenotypes that have been registered for patients suffering from Limb Girdle Muscular Dystrophy 2I (LGMD2I) and Facioscapulohumeral Muscular Dystrophy (FSHD) diseases to answer this question. The SPARQL query is as follows:

 
PREFIX doid: <http://purl.obolibrary.org/obo/doid#>
 
PREFIX ogdi: <http://purl.bioontology.org/ontology/OGDI#>
 
PREFIX omim: <http://purl.bioontology.org/ontology/OMIM/>
 
SELECT DISTINCT ?phenotype {
 
 SERVICE <**FKRP-REGISTRY-SPARQL**-**ENDPOINT**>
   
{ ?patient_FKRP ogdi:hasDisease omim:607155.
   
   ?patient_FKRP doid:has_symptom ?phenotype }
 
 SERVICE <**FSHD-REGISTRY-SPARQL-ENDPOINT**>
   
{ ?patient_FSHD ogdi:hasDisease omim:158900.
   
   ?patient_FSHD doid:has_symptom ?phenotype }
 
 FILTER (isURI(?phenotype))
 
  }

In our case, the result of this query returned the respective shared phenotypes for both diseases: “fatigue” (obo:HP_0012378), “muscle weakness” (obo:HP_0001324), and “rigidity” (obo:HP_0002063).

The same occurs for questions (4) and (5) as we have collected information regarding patients' diagnosis. This information was integrated according to the specifications for each disease. However, we are able to cross information between therapies and diseases due to our standardization strategy based on community-shared and common ontologies in all patient registries. For instance, if we query the different registries looking for patients treated with “ACE inhibitors” (i.e., ndfrt:N0000029130), we can easily find a correlation between DM (omim:160900) and LGMD2I (omim:607155) diseases:

 
PREFIX snomedct: <http://purl.bioontology.org/ontology/SNOMEDCT/>
 
PREFIX ogdi: <http://purl.bioontology.org/ontology/OGDI#>
 
PREFIX ndfrt: <http://purl.bioontology.org/ontology/NDFRT/>
 
SELECT DISTINCT ?disease WHERE {
 
 { SERVICE <**EHDN-REGISTRY-SPARQL-ENDPOINT**>
   
{?patient_EHDN snomedct:uses_substance ndfrt:N0000029130.
   
  ?patient_EHDN ogdi:hasDisease ?disease}  }
 
 UNION {SERVICE <**DM-REGISTRY-SPARQL-ENDPOINT**>
   
{?patient_DM snomedct:uses_substance ndfrt:N0000029130.
   
  ?patient_DM ogdi:hasDisease   ?disease}  }
 
 UNION {SERVICE <**FKRP-REGISTRY-SPARQL-ENDPOINT**>
   
{?patient_FKRP snomedct:uses_substance ndfrt:N0000029130.
   
  ?patient_FKRP ogdi:hasDisease   ?disease}  }
 
 UNION {SERVICE <**FSHD-REGISTRY-SPARQL-ENDPOINT**>
   
{?patient_FSHD snomedct:uses_substance ndfrt:N0000029130.
   
  ?patient_FSHD ogdi:hasDisease   ?disease}  }
 
  }

However, answering questions such as (6) is very complex. The difficulty resides in finding structured patient's states of morbidity in each registry. Diseases states are usually stored and described as long plain-text fields without suitable structure, which makes the task of finding similarities in that information more complex. Additionally, not all patient registries have this type of information, creating barriers to crossing information among different registries. Regarding that, we do not integrate different states of morbidity of each patient into our system.

In contrast, the two following questions, (7) and (8), can be more easily answered due to the structured information available regarding different diseases and respective patient phenotypes. To give an example for question (7), we can randomly choose phenotypes such as “fatigue” (obo:HP_0012378) and “muscle weakness” (obo:HP_0001324) and simply count how many patients share both:

 
PREFIX doid: <http://purl.obolibrary.org/obo/doid#>
 
PREFIX obo: <http://purl.obolibrary.org/obo/>
 
SELECT (COUNT(DISTINCT ?patient) as ?count) WHERE {
 
 { SERVICE <**EHDN-REGISTRY-SPARQL-ENDPOINT**>
   
{?patient doid:has_symptom obo:HP_0012378.
   
  ?patient doid:has_symptom obo:HP_0001324 }  }
 
 UNION {SERVICE <**DM-REGISTRY-SPARQL-ENDPOINT**>
   
{?patient doid:has_symptom obo:HP_0012378.
   
  ?patient doid:has_symptom obo:HP_0001324 }  }
 
 UNION {SERVICE* < ***FKRP-REGISTRY-SPARQL-ENDPOINT**>
   
{?patient doid:has_symptom obo:HP_0012378.
   
  ?patient doid:has_symptom obo:HP_0001324 }  }
 
 UNION {SERVICE <**FSHD-REGISTRY-SPARQL-ENDPOINT**>
   
{?patient doid:has_symptom obo:HP_0012378.
   
  ?patient doid:has_symptom obo:HP_0001324 }  }
 
  }

Using this schema allows us to find up to forty-one patients spread along the different databases. To answer question (8), we can also make a federated query to all registries to retrieve a list of associations between phenotypes and diseases. Given that, we are able to detect the most common associations by counting the number of patient's occurrences, in which the phenotype-disease association was identified:

 
PREFIX doid: <http://purl.obolibrary.org/obo/doid#>
 
PREFIX ogdi: <http://purl.bioontology.org/ontology/OGDI#>
 
SELECT ?phen ?disease (COUNT(DISTINCT ?patient) as ?count) WHERE {
 
 { SERVICE <**EHDN-REGISTRY-SPARQL-ENDPOINT**>
   
{?patient doid:has_symptom ?phen.
   
  ?patient ogdi:hasDisease ?disease }  }
 
 UNION {SERVICE <**DM-REGISTRY-SPARQL-ENDPOINT**>
   
{?patient doid:has_symptom ?phen.
   
  ?patient ogdi:hasDisease ?disease }  }
 
 UNION {SERVICE <**FKRP-REGISTRY-SPARQL-ENDPOINT**>
   
{?patient doid:has_symptom ?phen.
   
  ?patient ogdi:hasDisease ?disease }  }
 
 UNION {SERVICE <**FSHD-REGISTRY-SPARQL-ENDPOINT**>
   
{?patient doid:has_symptom ?phen.
   
  ?patient ogdi:hasDisease ?disease }  }
 
 FILTER (isURI(?phen))
 
 FILTER (isURI(?disease))
 
  } GROUP BY ?phen ?disease ORDER BY ?count

By querying our system, we are able to detect, for instance, that phenotypes such as “Muscular weakness” (HP:0001324) and “Fatigue” (HP:0012378) are more common in MUSCULAR DYSTROPHY (OMIM:607155) diseases and phenotypes such as “Myotonia” (HP:0002486) and “Fatigue” (HP:0012378) are more representative in MYOTONIC DYSTROPHY 1 (OMIM:160900) diseases.

## 5. Discussion

The IRDiRC (International Rare Diseases Research) consortium defined several overarching objectives, to achieve until 2020 [40]. Some of these goals include, for instance, to make data accessible to the research community or to promote tools and standards that simplify networking between data centres.* *The present solution was built upon these general needs, offering an opportunity to access patients distributed data in a common Web platform. The semantic layer approach offers a technological solution that enables data and metadata sharing, following common ontologies and standards, as described in the whole document. In our research work, we identified how these Semantic Web technologies can be tailored to the patient registries integration scenario. Although our results are successful, they highlight two major issues.

First, identifying the proper common ontology to be used across patient registries is a cumbersome challenge. While COEUS empowers this process at the technical level, there still has to be an agreement between stakeholders on what ontologies will be used and how will their data be properly mapped to them. This introduces a new challenge, as distinct ontologies need to be adequately mapped [41]. In this point, it is important to highlight that the creation of mappings between patient registries elements and ontologies is a critical point for data quality and reliability.

Rare diseases registry researchers frequently need to extract the primary clinical information and translate it into the registry data elements. This process is the key for the validity of outcomes that are under the scope of the registry. Both phenotypic information and final diagnosis have to be derived from the clinical examination, genetic, histopathological, and other laboratory tests, and radiological images, among some other specific sources, which are all challenging due to their heterogeneity and complexity.

In addition, standardization of the primary sources of information is an important issue for registries, but, in some situations, it is not possible. The translational process from the real clinical status of the patient to the information saved and stored into the registry database implies some potential risk of introducing some bias information. The establishment of mappings between information based on ontological terms could lead us to obtaining standardized data, but not valid results. Thus, when phenotypic data are not well defined or it is incorrectly translated into the database elements, this phenotypic information might be linked to wrong ontological terms. Likewise, ontological terms are not always as comprehensive as free text and, therefore, the ability of an ontology to cover all phenotypic traits of specific diseases is another limitation factor. In this regard, the collaboration with ontology developers in order to expand with further ontological terms and, hence, to align ontology representations with the current knowledge is needed. This active translational dialogue among the actors in the clinical and research domains is important both to stimulate the use of standards in patient registries and to ensure an appropriate description of the current domain knowledge in biomedical ontologies. In this challenging scenario, the mapping of clinical terms has to be undertaken according to quality procedures.

Nevertheless, several organizations are publishing common data element models in order to solve the interoperability problem among different patient registries. Although these efforts ensure interoperability within the selected domain, interoperability across application domain boundaries is not automatically possible [42].

Furthermore, there are over 600 rare disease registries in Europe alone, the majority not currently using a specific ontology. Although there is an overall desire in the community to increase harmonization, there is a lack of time and resources to change established procedures.

Second, convincing data owners of the true value of sharing their registry data is a cumbersome task. In addition to the privacy and security issues, data owners fail to realize the incentives underlying the sharing of their data. To overcome this in the future, financing projects should include clear guidelines to mandate the anonymous sharing of data for research purposes. Including these political policies would shed a new light on the benefits of sharing rare disease patient data with a broader community, truly unlocking its potential.

## 6. Conclusions

This work introduces a Semantic Web based layer that provides a holistic perspective over the wealth of knowledge stemming from linked patient registries supported by the growing number of research projects.

Our results are significant in at least three major respects: (1) the use of a model agnostic system, which enables the mapping of patient registries' data from any format to a common shared ontology; (2) the creation of an independent system that can be plugged into any existing patient registry without changing it; this enables the extraction of relevant data elements while maintaining patients' data privacy and security; (3) the adoption of Semantic Web technologies to promote a better translation, interpretation, federation, and discovery of new knowledge acquired from linked patient registries datasets.

Finally, this solution enables performing distributed queries to a federated system of linked patient registries. As a result, researchers can easily access a broad set of patient registries just like they would access a single system. We believe this is a milestone towards semantic interoperable rare diseases knowledge and will bring us one step closer to personalized medicine.
